# Draft Genome Sequence of a Novel Rhabdovirus Isolated from Deinocerites Mosquitoes

**DOI:** 10.1128/genomeA.01438-17

**Published:** 2018-05-10

**Authors:** Connie L. Xu, Paul G. Cantalupo, Maria Teresa Sáenz-Robles, Amy Baldwin, Dan Fitzpatrick, Douglas E. Norris, Ethan Jackson, James M. Pipas

**Affiliations:** a Department of Biological Sciences, University of Pittsburgh, Pittsburgh, Pennsylvania, USA; b AU/UGA Medical Partnership, University of Georgia, Athens, Georgia, USA; c Department of Molecular Microbiology and Immunology, Johns Hopkins Malaria Research Institute, Baltimore, Maryland, USA; d Department of Pathobiology, St. George’s University, St. George, Grenada; e Microsoft Research, Redmond, Washington, USA

## Abstract

We present a draft genome of a novel rhabdovirus, called Grenada mosquito rhabdovirus 1 (GMRV1), with homology to Wuhan mosquito virus 9 (WMV9) (NCBI reference sequence NC_031303), isolated from Deinocerites mosquitoes. The genome has a length of 14,420 nucleotides and encodes five open reading frames.

## GENOME ANNOUNCEMENT

Members of the Rhabdoviridae family are enveloped negative-sense single-stranded RNA viruses. They are known to infect a broad range of hosts, including plants, mammals, and insects (1). Rhabdovirus genomes contain five key structural proteins, a nucleocapsid protein N, phosphoprotein P, matrix protein M, glycoprotein G, and polymerase L, as well as 3′ leader and 5′ trailer sequences. The sizes of the genomes range from 11 kb to 15 kb.

Grenada mosquito rhabdovirus 1 (GMRV1) was recovered from a pool of 29 female Deinocerites sp. mosquitoes captured near Black Bay Beach in St. John Parish on the island of Grenada (12.12°N, 61.75°W) in March 2015. RNA was extracted from the pool of mosquitoes with TRIzol LS reagent (Ambion) following the manufacturer's instructions. rRNA (human-mouse-rat) was depleted from the sample, and a next-generation sequencing (NGS) library was prepared with the IntegenX RNA-Seq directional kit for Illumina. The library was sequenced at the Harvard Medical School BioPolymers Facility using HiSeq rapid sequencing to generate 100-bp paired-end reads.

We used Pickaxe, an in-house virus discovery software, on the set of 287 million sequence reads (2). After subtraction of sequences with an alignment to *Culicidae* and human sequences in GenBank, 90 million nonhost reads remained. Using CLC Assembly Cell (Qiagen), Pickaxe assembled the 78 million nonhost reads into 34,956 contigs that were subsequently annotated using a BLAST+/RAPSearch pipeline (3, 4).

A 14,420-nucleotide (nt) contig was initially identified as a rhabdovirus from a RAPSearch alignment with 46% identity to the RNA-dependent RNA polymerase (RdRp) of Wuhan mosquito virus 9 (WMV9) (NCBI reference sequence NC_031303). GMRV1 contains five open reading frames (ORFs), a 151-nt 3′ leader, and a 356-nt 5′ trailer. The 3′ leader and 5′ trailer show no complementarity. ORFs 1 to 5 are located at nt 152 to 1699, nt 1847 to 3256, nt 3375 to 4274, nt 4454 to 6397, and nt 7192 to 14064, respectively. We predict these ORFs to encode the N, P, M, G, and L proteins based on BLASTP searches and the genomic layout of Rhabdoviridae. The nucleoprotein N shares 37% identity with ORF1 in WMV9, the matrix protein M shares 22% identity with ORF3 in WMV9, the glycoprotein G shares 30% identity with the glycoprotein in WMV9, and the polymerase protein L shares 47% identity with the RdRp in WMV9. A BLASTP search of the predicted phosphoprotein P did not produce any homologous virus proteins.

RNA virus phylogeny was recently expanded through deep sequencing of metagenomes (5, 6). Given the large phylogenetic distance of GMRV1 to WMV9 and the limited number of other viral matches to its ORFs, this finding expands the known diversity of negative-sense single-stranded RNA [ssRNA(−)] viruses.

### Accession number(s).

The genome sequence of GMRV1 was deposited in GenBank under the accession number MG385079.
